# Expression of Ku70 correlates with survival in carcinoma of the cervix

**DOI:** 10.1054/bjoc.2000.1510

**Published:** 2000-12

**Authors:** C R Wilson, S E Davidson, G P Margison, S P Jackson, J H Hendry, C M L West

**Affiliations:** CRC Experimental Radiation Oncology, Carcinogenesis Groups, Department of Clinical Oncology, Christie Hospital (NHS) Trust, Wilmslow Road, Manchester, M20 4BX; CRC Institute of Cancer and Developmental Biology, Cambridge University, Tennis Court Road, Cambridge, CB2 1QR, UK

**Keywords:** DNA repair, Ku, radiosensitivity, immunohistochemistry, DNA-PK, predictive assay

## Abstract

Cervical carcinoma affects around 3400 women in the UK each year and advanced disease is routinely treated with radiation. As part of a programme to establish rapid and convenient methods of predicting tumour and patient responses to radiotherapy, we have examined the relationship between the pre-treatment expression of the Ku components of the DNA damage recognition complex DNA-PK and patient survival in cervical carcinoma. Using immunohistochemistry of formalin-fixed sections of tumour biopsies, antibodies to Ku70 and Ku80 stained identical regions of tumour and there was a high degree of correlation between the mean number of cells stained positive for the two components in 77 tumours (r = 0.82, *P*< 0.001). In 53 tumours there was a borderline significant correlation between measurements of tumour radiosensitivity (surviving fraction at 2 gray: SF2) and Ku70 expression (r = 0.26, *P* = 0.057) and no correlation for Ku80 (r = 0.18, *P* = 0.19). However, all tumours with a low number of Ku70 or Ku80 positive cells were radiosensitive. Furthermore, using log-rank analysis there was significantly higher survival in the patients whose tumours had a low Ku70 expression (*P* = 0.046). This difference was also reflected with Ku80, but did not reach statistical significance (*P* = 0.087). The study suggests that lack of Ku protein leads to radiosensitivity in some tumours and that other factors are responsible for radiosensitive tumours with high Ku expression. It is likely that the most accurate prediction of treatment outcome will lie in assessing the expression of several proteins involved in the recognition and repair of DNA damage, one of which will be Ku. © 2000 Cancer Research Campaign http://www.bjcancer.com


*British Journal of Cancer* (2000) **83**(12), 1702–1706
© 2000 Cancer Research Campaign
doi: 10.1054/ bjoc.2000.1510, available online at http://www.idealibrary.com on IDEAL®

### Expression of Ku70 correlates with survival in carcinoma of the cervix

**CR Wilson[1], SE Davidson[2], GP Margison[3], SP Jackson[4], JH Hendry[1] and CML West[1]**

CRC [1]Experimental Radiation Oncology and [3]Carcinogenesis Groups and [2]Department of Clinical Oncology, Christie Hospital (NHS) Trust, Wilmslow Road, Manchester, M20 4BX; [4]CRC Institute of Cancer and Developmental Biology, Cambridge University Tennis Court Road, Cambridge, CB2 1QR, UK

**Summary** Cervical carcinoma affects around 3400 women in the UK each year and advanced disease is routinely treated with radiation. As part of a programme to establish rapid and convenient methods of predicting tumour and patient responses to radiotherapy, we have examined the relationship between the pre-treatment expression of the Ku components of the DNA damage recognition complex DNA-PK and patient survival in cervical carcinoma. Using immunohistochemistry of formalin-fixed sections of tumour biopsies, antibodies to Ku70 and Ku80 stained identical regions of tumour and there was a high degree of correlation between the mean number of cells stained positive for the two components in 77 tumours (r = 0.82, $P < 0.001$). In 53 tumours there was a borderline significant correlation between measurements of tumour radiosensitivity (surviving fraction at 2 gray: SF2) and Ku70 expression (r = 0.26, $P = 0.057$) and no correlation for Ku80 (r = 0.18, $P = 0.19$). However, all tumours with a low number of Ku70 or Ku80 positive cells were radiosensitive. Furthermore, using log-rank analysis there was significantly higher survival in the patients whose tumours had a low Ku70 expression ($P = 0.046$). This difference was also reflected with Ku80, but did not reach statistical significance ($P = 0.087$). The study suggests that lack of Ku protein leads to radiosensitivity in some tumours and that other factors are responsible for radiosensitive tumours with high Ku expression. It is likely that the most accurate prediction of treatment outcome will lie in assessing the expression of several proteins involved in the recognition and repair of DNA damage, one of which will be Ku. © 2000 Cancer Research Campaign http://www.bjcancer.com

**Keywords:** DNA repair; Ku; radiosensitivity; immunohistochemistry; DNA-PK; predictive assay

DNA-dependent protein kinase (DNA-PK) is a serine threonine kinase whose activity is dependent on DNA binding. It is composed of a catalytic subunit, DNA-PKcs, and the regulatory subunit Ku (Smith and Jackson, 1999). The latter is a heterodimer composed of ~70 kDa (Ku70) and ~80kDa (Ku80) proteins. There are a number of observations that suggest that DNA-PK has a specific role in the repair of DNA double strand breaks. Embryonic stem cells lacking Ku70 or Ku80 are hypersensitive to ionizing radiation and are deficient in V(D)J recombination, a process that requires specific formation and rejoining of double-strand breaks (Gu et al, 1997; Nussenzweig et al, 1997). Also, when Chinese hamster ovary *xrs-6* cells lacking functional Ku80 are transfected with the human chromosomal fragment coding for Ku80, V(D)J recombination and radiation sensitivity are restored to normal levels (Peterson et al, 1995; Ross et al, 1995). Finally, irradiation of MCF-7 or Hela cell lines in the presence of wortmannin (a phosphatidylinositol 3-kinase ($PI_3$ kinase) inhibitor) increases sensitivity to ionizing radiation 3–5 fold (Price and Youmell, 1996). The latter finding has been attributed to the down-regulation of DNA-PK activity by wortmannin (Boulton et al, 1996), and is thought to be due to the homology between $DNA\text{-}PK_{cs}$ and $PI_3$ kinase (Hartley et al, 1995; Poltoratsky et al, 1995; Izzard et al, 1999).

There is evidence that the intrinsic radiosensitivity of tumour cells is a determinant of outcome following radiotherapy (West et al, 1997). This has led to interest in finding a method for measuring tumour radiosensitivity that is applicable for wide-spread clinical use. The immunohistochemical analysis of DNA repair enzymes involved in the recognition or repair of radiation-induced DNA damage may have potential as a predictive assay of tumour radiosensitivity. In support of this, we have already shown that the expression of the human endonuclease HAPI correlates with the radiation sensitivity of human cervix tumours (Herring et al, 1998). In this study we have examined the possible role of Ku protein in determining tumour radiosensitivity and response to radiotherapy.

*Received 12 May 2000*
*Revised 14 August 2000*
*Accepted 16 August 2000*

*Correspondence to:* C West

### MATERIALS AND METHODS

### Immunohistochemistry of Ku70 and Ku80

Formalin-fixed, paraffin embedded sections (4 μm thick) were dewaxed for 10 min in xylene and rehydrated by passage through a graded ethanol series to tap water. Endogenous peroxidases were blocked with 3% hydrogen peroxide in Tris buffered saline (TBS) for 20 min. After washing in TBS for 5 min, the sections were blocked for a further 30 min using 10% normal swine serum (Dako) in TBS. Serum was removed by tapping and 100 μl of antibody or TBS for control sections was added. Anti-Ku70 and -Ku80 rabbit polyclonal antisera were raised against bacterially expressed full length human Ku70 or Ku80. They were used as 1:500 and 1:1000 dilutions in TBS, respectively. After incubation at 4˚C for 18 hours in TBS, the sections were washed 3 times in TBS for 5 min and incubated at room temperature for 30 min with 100 μl of biotinylated swine anti-rabbit IgG heavy chain and light chain antiserum (SARBO, Dako) at 1:400 in TBS. The sections were then washed 3 times in TBS for 5 min and incubated for 30 min at room temperature with 100 µ1 biotinylated peroxidase-streptavidin complex (ABC, Dako). Following three 5 min washes in TBS, the sections were stained for 5 min with 100 µl DAB (1 fast-DAB tablet (Sigma) dissolved in 20 ml TBS plus 10 µl 30% $H_2O_2$). The DAB was removed and the sections washed in running tap water for 10 min and counterstained in Gills haemotoxylin for 1 min and blue alkali for 1 min. The sections were then dehydrated through the graded ethanol series to xylene and mounted.

### Quantification of Ku70 and Ku80 expression in tumour sections using light microscopy

The number of cells in tumour samples stained positive for Ku70 and Ku80 was determined by scoring ten microscope fields of 100 tumour cells, without prior knowledge of radiosensitivity and treatment outcome, and determining the percentage of cells positive for Ku70 and Ku80 expression. The values are expressed as mean percentages of the 10 fields plus or minus the standard error of the mean.

### Tumour radiosensitivity

The study was performed following South Manchester Medical Research Ethics Committee approval. The patient details, treatment protocols and assay methods are described elsewhere (West et al, 1997). Briefly, all patients had bulky advanced disease and received radiotherapy with curative intent. Small volume disease was treated with intracavitary low-dose rate caesium alone. Remaining patients either received external beam irradiation (16 2 Gy fractions in 3 weeks) followed by two intracavitary caesium insertions or external beam over 4 weeks supplemented by a single low-dose rate intracavitary insertion. Random cervical punch biopsies were taken prior to initiation of treatment. Radiosensitivity was determined in vitro using a soft agar clonogenic assay as sensitivity to a single dose of 2 Gy radiation (SF2). Follow-up schedules of 3 monthly in the first 2 years, 4 monthly in the third year and 6 monthly for the next 2 years were employed. Local recurrence within the radiation field was confirmed histologically and/or using radiological techniques. Disease-specific survival was measured with minimum and median follow-up times of 24 and 58 months, respectively.

### DATA ANALYSIS

The SPSS statistical software program was used to test for correlations between quantitative variables by the establishment of non-parametric linear regression. The probabilities of overall survival and local control was determined using univariable log-rank analysis, with the variables grouped into two (above and below median values) bands. As group boundaries correspond to medians, values falling on the boundaries meant that, in practice, the data subsets were not always exactly the same size. A significance level of 0.05 was used throughout.

### RESULTS

The specificity of the antibodies was investigated in western blots of human whole cell extracts, where the Ku70 antibody recognized a single 70 kDa protein, and the Ku80 antibody recognized a single 80 kDa protein. Tris buffered saline (TBS) was used as a negative control. No staining was seen in any of the sections when TBS was used. Staining for Ku70 and Ku80 was predominantly nuclear.

To determine whether the scoring was reproducible, the 77 sections were scored blind twice and a *t*-test performed on the data. There was no significant difference between the percentage of cells stained positive for the proteins in the two data sets. Also the repeat scoring results were highly correlated for both Ku70 (r = 0.96, $P < 0.001$) and Ku80 (r = 0.98, $P = 0.001$). In order to examine inter-observer reproducibility, 12 sections were scored independently by another individual (CMLW). There was a significant correlation between the percentage of cells scored positive for both Ku70 (r = 0.74, $P = 0.006$) and Ku80 (r = 0.62, $P = 0.031$).

The percentage of cells expressing Ku70 and Ku80 in different tumours ranged from 0–95% for both (Table 1). Several of the sections exhibited pronounced intercellular variation in staining for Ku70 and Ku80 and a similar pattern of staining for both Ku70 and Ku80 could be seen in the individual sections. There was a high degree of correlation between the percentage of cells expressing Ku70 and Ku80 in the tumour sections and 11 of the 77 tumours had a low number of cells expressing Ku proteins (Figure 1). For some of the tumours data were available for mitotic ($n = 37$) and Ki67 ($n = 25$) index. There were no correlations between tumour

**Table 1** Biological parameters

| | *n* | Mean ± SD | Median | Range |
|---|---|---|---|---|
| Ku70[a] | 77 | 67 ± 22% | 75% | 0–95% |
| Ku80[a] | 77 | 69 ± 24% | 77% | 0–95% |
| SF2[b] | 53 | 0.45 ± 0.18 | 0.42 | 0.14–0.91 |

[a]The levels of Ku70 and Ku80 expression was determined by scoring ten microscope fields of 100 tumour cells and determining the percentage of cells positive for Ku70 and Ku80 expression. Values were expressed as mean percentages of the 10 fields scored. The mean, median and range of the mean values for the 77 tumours scored are given. [b]SF2 is surviving fraction at 2 Gy, a measure of tumour radiosensitivity.

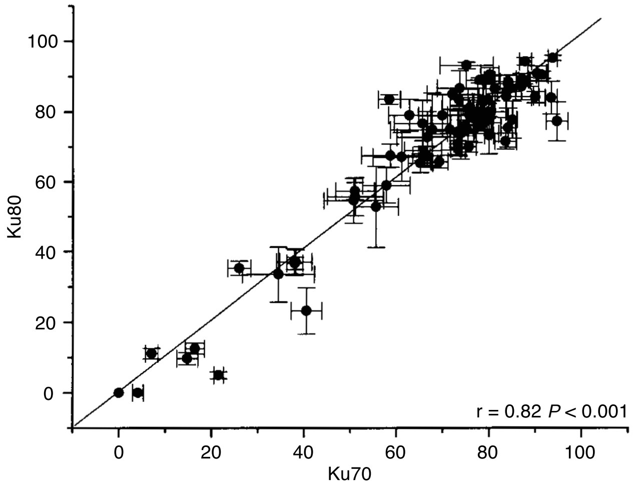

**Figure 1** Ku70 and Ku80 expression in adjacent sections of 77 cervix carcinomas. Data points represent the mean and the standard error of the percentage of cells stained positive for Ku70 or Ku80 in 10 microscope fields of 100 tumour cells

© 2000 Cancer Research Campaign

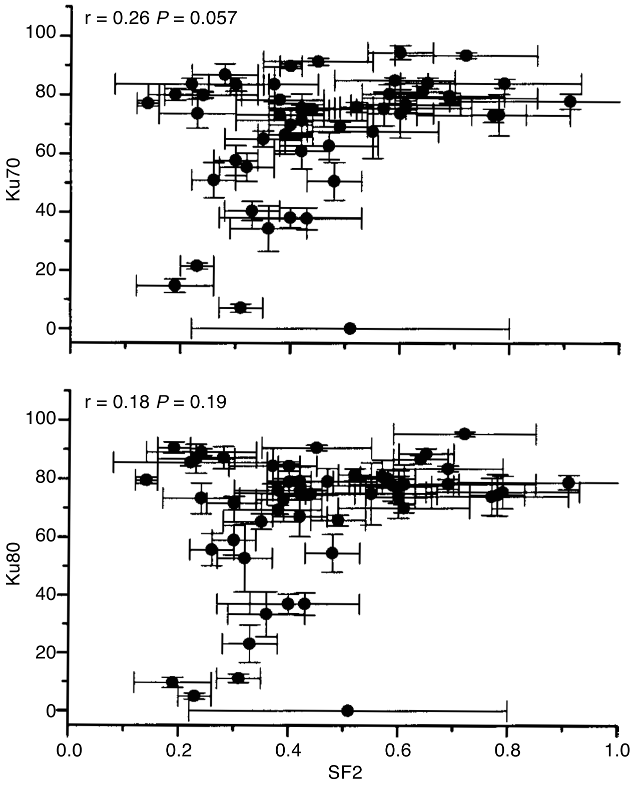

**Figure 2** Relationship between SF2 and Ku70 (top) and Ku80 (bottom) expression in 53 cervix carcinomas. Data points represent the mean with standard errors of 10 microscope fields of 100 tumour cells (Ku expression) or 4–8 replicate colony counts (SF2)

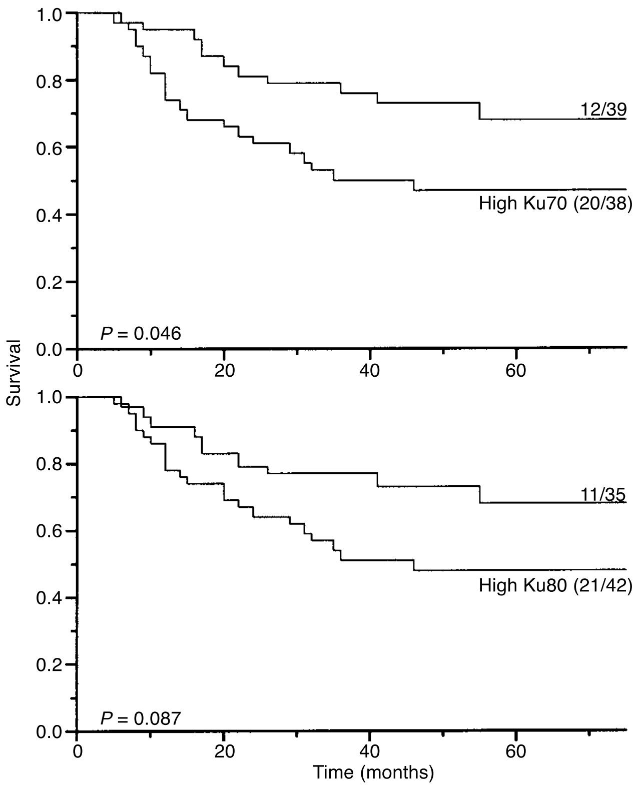

**Figure 3** Survival following radiotherapy versus Ku70 (top) and Ku80 (bottom) expression. The data from 77 patients were stratified according to the median of the mean number of cells in tumour samples stained positive for the Ku proteins. Upper arms represent patients with low tumour expression of Ku70 or Ku80. The number of deaths/total number of patients per group is given on each arm

**Table 2** Ku70 and Ku80 expression in relation to clinical parameters

| | | *n* | Ku70 (%) | Ku80 (%) |
|---|---|---|---|---|
| Stage | I | 15 | 67 | 73 |
| | II | 29 | 73 | 73 |
| | III | 33 | 76 | 81 |
| Grade | WELL | 14 | 70 | 74 |
| | MOD | 39 | 69 | 77 |
| | POOR | 8 | 69 | 78 |
| Histology | SCC | 61 | 69 | 76 |
| | ADENO | 11 | 64 | 68 |
| Age | <55 yrs | 32 | 76 | 78 |
| | ≥55 yrs | 45 | 73 | 76 |

Values are the mean of the mean percentage of cells in tumour samples stained positive for the Ku proteins.

proliferation and the expression of either Ku70 or Ku80 ($P > 0.26$ for all).

SF2 values were available for 53 of the tumour samples (West et al, 1997) and these ranged from 0.14 to 0.91 with a median value of 0.42 (Table 1). There was a borderline significant correlation between intrinsic sensitivity to ionizing radiation and Ku70 expression ($r = 0.26$, $P = 0.057$) but no relationship for Ku80 ($r = 0.18$, $P = 0.19$). These relationships are illustrated in Figure 2 and show that tumours with high SF2 value (i.e., radioresistant tumours with SF2 > 0.60) all had a high percentage of Ku positive cells.

Table 2 summarizes the distribution of patients regarding disease stage, tumour grade and histology. The median age for the 77 patients was 55 years (range 29–79 years). Relationships were examined between Ku expression and the clinical parameters listed in Table 2. No significant relationships were seen. Log-rank analysis was performed to determine whether Ku70 or Ku80 expression could be used as prognostic indicators for the response of cervical carcinoma patients to radiotherapy (Figure 3). Survival levels for patients with tumours with high versus low Ku70 were 47% and 69%, respectively (Table 3). The difference reached statistical significance ($P = 0.046$). There was also a higher level of survival for patients whose tumours had a below median percentage of Ku80 positive cells (69%) as opposed to those with higher than median level (50%), but the differences did not reach statistical significance ($P = 0.087$). Similar relationships were seen for local control but these also did not reach statistical significance (Figure 4). Table 3 summarizes the outcome data for Ku70, Ku80, SF2, age and stage for the patients included in the study. Although, there were insufficient patient numbers for a multivariate analysis, bivariable analyses were carried out. Borderline significant correlations were seen after allowing for age and stage but the prognostic significance of Ku expression was lost after allowing for SF2. Also while there appeared to be no stage-dependent effect, there was for age. Tumour Ku expression was not important for young women, below the median age. However, for older women there was a large difference in survival for both Ku70 ($P = 0.040$) and Ku80 ($P = 0.0034$).

© 2000 Cancer Research Campaign

**Table 3** Outcome data

| Parameter | Stratification | Survival | | Local control | |
|---|---|---|---|---|---|
| Ku70 | <75% | 69% | | 82% | |
| | >75% | 47% | *0.046* | 71% | *0.19* |
| Ku80 | <77% | 69% | | 85% | |
| | >77% | 50% | *0.087* | 69% | *0.060* |
| SF2 | <0.42 | 78% | | 85% | |
| | >0.42 | 36% | *0.0009* | 68% | *0.079* |
| Stage | I | 93% | | 93% | |
| | II | 55% | | 76% | |
| | III | 45% | *0.0036* | 70% | *0.082* |
| Age | <55 yrs | 50% | | 65% | |
| | >55 yrs | 64% | *0.29* | 84% | *0.067* |

Values in italics are *P* values from log-rank analyses.

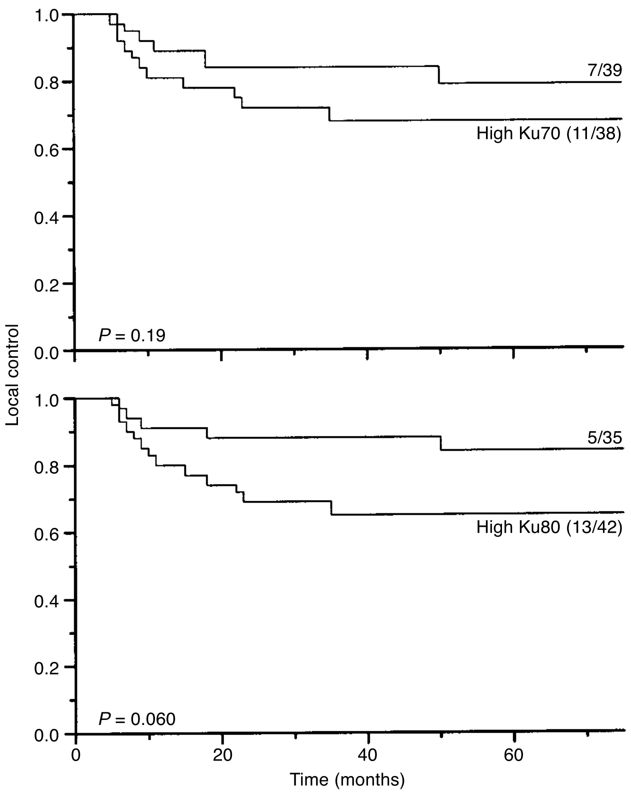

**Figure 4** Local control following radiotherapy versus Ku70 (top) and Ku80 (bottom) expression. The data from 77 patients were stratified according to the median number of cells in tumour samples stained positive for the Ku proteins. Upper arms represent patients with low tumour expression of Ku70 or Ku80. The number of deaths/total number of patients per group is given on each arm

### DISCUSSION

Staining for Ku70 and Ku80 in all the cervix tumours studied was predominantly nuclear as expected from previous reports using direct immunofluorescence (Higashiura et al, 1992; Hong et al, 1994). There was a high degree of intratumour heterogeneity in Ku expression. This is unlikely to result from heterogeneity in cell cycle phase distribution for several reasons. First, Ku has been shown to remain in the nucleus throughout the cell cycle (Yaneva and Jhiang, 1991). Second, no correlation was seen between Ku70 or Ku80 expression and tumour proliferation measured as either mitotic or Ki67 index. Also, there was a good correlation between the number of cells in tumour samples stained positive for Ku70 and Ku80 that supports in vitro studies of the interdependence of the two heterodimer components (Satoh et al, 1995). The latter suggests that intratumour heterogeneity in Ku expression is not a staining artefact.

In a study from which the cohort of patients studied here was taken, tumour SF2 has been shown to be a highly significant prognostic factor for cervical carcinoma undergoing radiotherapy (West et al, 1997, 1998). SF2 is a measure of the intrinsic radiosensitivity of cells. As described earlier, there is ample evidence for a role of Ku in determining cellular response to radiation. However, no significant correlation was found between SF2 and the expression of Ku70 or Ku80. A similar lack of correlation between SF2 and DNA-PK activity has been shown, albeit in cell lines derived from 9 malignant gliomas (Allalunis-Turner et al, 1995). Others have shown that, in comparison with radioresistant lines, some radiosensitive tumour cell lines have less DNA-PK activity (Polischouk et al, 1999; Sirzen et al, 1999). There has been one other study using human tumours where SF2 has been measured as described here (Bjork Eriksson et al, 1999). The latter study, on head and neck tumours, also showed no significant correlation between SF2 and either DNA-PKcs ($r = 0.22$, $P = 0.081$) or Ku (p70/p80) ($r = 0.064$, $P = 0.62$) expression in 64 tumours. However, in both the latter study and ours, tumours with a low percentage of Ku or DNA-$PK_{cs}$ positive cells were all sensitive, with low SF2 values. This suggests that loss of Ku expression may account for the radiosensitivity of some tumours. However, the radiosensitivity of tumours with a high percentage of Ku positive cells may be due to the mutation or down-regulation of other DNA-damage sensing or repair proteins. Alternatively/additionally the lack of correlation seen may be because studies using primary human tumours or early passage cell lines assessing radiosensitivity using clonogenic assays will analyse a sub-population of cells whilst immunohistochemical staining/cell extract measurements are performed on the total tumour population. There may also be cell type-dependent differences.

A link between Ku expression and ionizing radiation sensitivity is further supported by the significantly higher survival for patients whose tumours had a low percentage of Ku70 positive cells ($P = 0.046$). The difference in patient outcome was also reflected with Ku80, but did not reach significance ($P = 0.086$). The poorer prognosis of patients with high Ku70 expression suggests that tumour cells with the ability to repair damaged DNA are more likely to survive and proliferate following treatment.

Interest in measuring radiosensitivity lies not only in tumours but also normal tissues (West et al, 1998). Therefore, it would be useful to examine the relationship between tumour and normal tissue expression levels of Ku proteins. No relationship was seen between DNA-PK mRNA levels/activity in a group of human fibroblast lines (Kasten et al, 1999). In addition, a recent study has shown that acquired radioresistance in a murine cell line was associated with an increase in Ku activity (Frit et al, 1999). It may be that the high levels of Ku protein seen in most of the tumours studied here are an acquired phenotype. In order to test this, parallel samples of tumour and normal tissue should be taken from patients.

In conclusion, despite reports of changes in radiosensitivity when Ku levels are markedly altered, no significant correlation was seen between tumour Ku protein expression and in vitro radiosensitivity. However, there was a wide variation in the degree

*© 2000 Cancer Research Campaign*

of antibody staining between tumours and all tumours with a low percentage of Ku positive cells were radiosensitive. In addition, we have shown that expression of Ku70 in human cervix carcinoma is prognostic for survival for patients undergoing radiotherapy. This finding suggests that DNA-PK is involved in determining cancer outcome via its involvement in DNA dsb repair but other factors are also important. It is likely that the most accurate prediction of treatment outcome will lie in assessing the expression of several proteins involved in the recognition and repair of DNA damage, one of which will be Ku.

### REFERENCES

Allalunis-Turner MJ, Lintott LG, Barron GM, Day RS 3rd and Lees Miller SP (1995) Lack of correlation between DNA-dependent protein kinase activity and tumor cell radiosensitivity. *Cancer Res* **55**: 5200–5202

Bjork Eriksson T, West C, Nilsson A, Magnusson B, Svensson M, Karlsson E, Slevin N, Lewensohn R and Mercke C (1999) The immunohistochemical expression of DNA-PKCS and Ku (p70/p80) in head and neck cancers: relationships with radiosensitivity. *Int J Radiat Oncol Biol Phys* **45**: 1005–1010

Boulton S, Kyle S, Yalcintepe L and Durkacz BW (1996) Wortmannin is a potent inhibitor of DNA double strand break but not single strand break repair in Chinese hamster ovary cells. *Carcinogenesis* **17**: 2285–2290

Frit P, Canitrot Y, Muller C, Foray N, Calsou P, Marangoni E, Bourhis J and Salles B (1999) Cross-resistance to ionizing radiation in a murine leukemic cell line resistant to cis-dichlorodiammineplatinum(II): role of Ku autoantigen. *Mol Pharmacol* **56**: 141–146

Gu Y, Seidl KJ, Rathbun GA, Zhu C, Manis JP, van der Stoep N, Davidson L, Cheng HL, Sekiguchi JM, Frank K, Stanhope Baker P, Schlissel MS, Roth DB and Alt FW (1997) Growth retardation and leaky SCID phenotype of Ku70-deficient mice. *Immunity* **7**: 653–655

Hartley KO, Gell D, Smith GC, Zhang H, Divecha N, Connelly MA, Admon A, Lees Miller SP, Anderson CW and Jackson SP (1995) DNA-dependent protein kinase catalytic subunit: a relative of phosphatidylinositol 3-kinase and the ataxia telangiectasia gene product. *Cell* **82**: 849–856

Herring CJ, West CM, Wilks DP, Davidson SE, Hunter RD, Berry P, Forster G, MacKinnon J, Rafferty JA, Elder RH, Hendry JH and Margison GP (1998) Levels of the DNA repair enzyme human apurinic/apyrimidinic endonuclease (APE1, APEX, Ref-1) are associated with the intrinsic radiosensitivity of cervical cancers. *Br J Cancer* **78**: 1128–1133

Higashiura M, Shimizu Y, Tanimoto M, Morita T and Yagura T (1992) Immunolocalization of Ku-proteins (p80/p70): localization of p70 to nucleoli and periphery of both interphase nuclei and metaphase chromosomes. *Exp Cell Res* **201**: 444–451

Hong TJ, Escribano J and Coca Prados M (1994) Isolation of cDNA clones encoding the 80-kd subunit protein of the human autoantigen Ku (p70/p80) by antisera raised against ciliary processes of human eye donors. *Invest Ophthalmol Vis Sci* **35**: 4023–4030

Izzard RA, Jackson SP and Smith GC (1999) Competitive and noncompetitive inhibition of the DNA-dependent protein kinase. *Cancer Res* **59**: 2581–2586

Kasten U, Plottner N, Johansen J, Overgaard J and Dikomey E (1999) Ku70/80 gene expression and DNA-dependent protein kinase (DNA-PK) activity do not correlate with double-strand break (dsb) repair capacity and cellular radiosensitivity in normal human fibroblasts. *Br J Cancer* **79**: 1037–1041

Nussenzweig A, Sokol K, Burgman P, Li L and Li GC (1997) Hypersensitivity of Ku80-deficient cell lines and mice to DNA damage: the effects of ionizing radiation on growth, survival, and development. *Proc Natl Acad Sci USA* **94**: 13588–13593

Peterson SR, Kurimasa A, Oshimura M, Dynan WS, Bradbury EM and Chen DJ (1995) Loss of the catalytic subunit of the DNA-dependent protein kinase in DNA double-strand-break-repair mutant mammalian cells. *Proc Natl Acad Sci USA* **92**: 3171–3174

Polischouk AG, Cedervall B, Ljungquist S, Flygare J, Hellgren D, Grenman R and Lewensohn R (1999) DNA double-strand break repair, DNA-PK, and DNA ligases in two human squamous carcinoma cell lines with different radiosensitivity. *Int J Radiat Oncol Biol Phys* **43**: 191–198

Poltoratsky VP, Shi X, York JD, Lieber MR and Carter TH (1995) Human DNA-activated protein kinase (DNA-PK) is homologous to phosphatidylinositol kinases. *J Immunol* **155**: 4529–4533

Price BD and Youmell MB (1996) The phosphatidylinositol 3-kinase inhibitor wortmannin sensitizes murine fibroblasts and human tumor cells to radiation and blocks induction of p53 following DNA damage. *Cancer Res* **56**: 246–250

Ross GM, Eady JJ, Mithal NP, Bush C, Steel GG, Jeggo PA and McMillan TJ (1995) DNA strand break rejoining defect in xrs-6 is complemented by transfection with the human Ku80 gene. *Cancer Res* **55**: 1235–1238

Satoh M, Wang J and Reeves WH (1995) Role of free p70 (Ku) subunit in posttranslational stabilization of newly synthesized p80 during DNA-dependent protein kinase assembly. *Eur J Cell Biol* **66**: 127–135

Sirzen F, Nilsson A, Zhivotovsky B and Lewensohn R (1999) DNA-dependent protein kinase content and activity in lung carcinoma cell lines: correlation with intrinsic radiosensitivity. *Eur J Cancer* **35**: 111–116

Smith GC and Jackson SP (1999) The DNA-dependent protein kinase. *Genes Dev* **13**: 916–934

West CM, Davidson SE, Roberts SA and Hunter RD (1997) The independence of intrinsic radiosensitivity as a prognostic factor for patient response to radiotherapy of carcinoma of the cervix [see comments]. *Br J Cancer* **76**: 1184–1190

West CM, Davidson SE, Elyan SA, Swindell R, Roberts SA, Orton CJ, Coyle CA, Valentine H, Wilks DP, Hunter RD and Hendry JH (1998) The intrinsic radiosensitivity of normal and tumour cells. *Int J Radiat Biol* **73**: 409–413

Yaneva M and Jhiang S (1991) Expression of the Ku protein during cell proliferation. *Biochim Biophys Acta* **1090**: 181–187

*© 2000 Cancer Research Campaign*
